# Description of the Distinctive Changes in the Colonic Microbiome Associated with Irritable Bowel Syndrome, Uncomplicated Diverticulitis, and Tubular Adenoma

**DOI:** 10.3390/biomedicines13102424

**Published:** 2025-10-03

**Authors:** Ramón Saavedra-Bravo, Alfonso Méndez-Tenorio, Mario Angel López-Luis, Eduardo Alejandro Dávila-Martínez, Marco Antonio Vázquez-Ávila, Lenin García-Gutierrez, Gloria León-Avila, Cindy Bandala, Mónica Alethia Cureño-Díaz, Verónica Fernández-Sánchez, José Antonio Morales-González, Eleazar Lara-Padilla, Javier Mancilla-Ramírez, Gabriela Ibáñez-Cervantes

**Affiliations:** 1 Sección de Estudios de Posgrado, Escuela Superior de Medicina, Instituto Politécnico Nacional, Mexico City 11340, Mexico; 2 Jefatura de Fomento y Divulgación Científica, Escuela Medico Naval, Secretaría de Marina, Mexico City 04830, Mexico; 3 Laboratorio de Biotecnología y Bioinformática Genómica, Departamento de Bioquímica, Escuela Nacional de Ciencias Biológicas, Instituto Politécnico Nacional, Mexico City 11340, Mexico; 4 Departamento de Zoología, Escuela Nacional de Ciencias Biológicas, Instituto Politécnico Nacional, Mexico City 11340, Mexico; 5 Laboratorio de Medicina y Neurociencia Traslacional, Escuela Superior de Medicina, Instituto Politécnico Nacional, Mexico City 11340, Mexico; 6 Instituto Nacional de Cancerología. Av. San Fernando No. 22, Col. Sección XVI Delegación Tlalpan, Mexico City 14080, Mexico; 7 Facultad de Estudios Superiores Iztacala, UNAM. Av. de los Barrios 1, Hab Los Reyes Ixtacala Barrio de los Árboles/Barrio de los Héroes, Tlalnepantla 54090, México; 8 Laboratorio de Medicina de Conservación, Escuela Superior de Medicina, Instituto Politécnico Nacional, Mexico City 11340, Mexico; 9 Laboratorio de Microbiología y Biología Molecular, Departamento de Desarrollo de Tecnologías, Centro Interdisciplinario de Ciencias Marinas, Instituto Politécnico Nacional, C. Instituto Politécnico Nacional s/n, La Paz 23096, Baja California Sur, Mexico

**Keywords:** acute irritable bowel syndrome (IBS), uncomplicated diverticulitis, tubular adenoma, microbiome, dysbiosis

## Abstract

**Background**: The pathogenesis of various colon-related pathologies, including irritable bowel syndrome, uncomplicated diverticulitis, and tubular adenoma, remains unknown, primarily due to their multifactorial nature. These gastrointestinal diseases are increasing in prevalence in Western countries and are common conditions worldwide. **Objective**: To identify intestinal microbiota signs and their associations with the development of colonic pathologies, such as irritable bowel syndrome, uncomplicated diverticulitis, and tubular adenoma. **Materials and Methods**: An observational, prospective, cross-sectional study was conducted to compare the microbiome among three conditions via 16S rRNA sequencing of biopsy samples obtained via colonoscopy. **Results**: The microbiome of individuals with tubular adenoma was less diverse than that of patients with diverticulitis and irritable bowel syndrome, with a lower abundance of commensal bacterial genera, such as *Catenibacterium*, *Bifidobacterium*, and *Faecalibacterium*, and an increase in several genera with known pathogenic roles, including *Escherichia–Shigella*, *Fusobacteria*, *Prevotella*, and *Haemophilus*. No significant association was found between the type of pathology and the total pathogenic or commensal disease score; however, a ratio of 2.54 to pathogenic/commensal was observed in the IBS patient group. In contrast, in the diverticulitis and adenoma patient groups, this ratio was 8. **Conclusions**: These results provide evidence supporting the proposal that alterations in the colonic microbiome could be involved in various colonic pathogeneses and that an imbalance between commensal and pathogenic populations could be directly related to pathogenesis in the microsystem. It is important to highlight the need for future studies.

## 1. Introduction

Previously, little attention has been given to the relationship between the normal colonic microbiome and its microhabitat. Several studies have shown the complexity of colonic homeostasis and the importance of the trillions of microorganisms that inhabit it [1].

The composition of the gut microbiome plays an essential role in inflammatory and metabolic diseases of the colon [2,3,4], and dysbiosis can be caused by a variety of factors, such as antibiotic use, motility disturbances, dietary modifications, and changes in gut physiology [5,6,7]. These factors can create a proinflammatory environment or allow pathogenic bacteria to overgrow [8,9]. Many disorders can affect the ability of the colon to function correctly. These include irritable bowel syndrome (IBS), diverticulitis, and adenomas, among others.

IBS is the most common functional gastrointestinal disorder in the Western world [10]. It is a multifactorial condition involving genetic, physiological, and psychological responses to stress, diet, age, geographic origin, infections, antibiotic use, immune-mediated intestinal injury, and microbiome dysbiosis [11]. In Mexico, it is estimated that between 16% and 30% of the population suffers from this syndrome, with a higher prevalence in women under 45 years of age. Its symptoms are related to altered gastrointestinal motility, hypersensitivity, immune activation, and changes in the composition and function of intestinal bacteria, as well as in the intestinal mucosal barrier [12]. To date, no single mechanism is known to explain the development of IBS.

Diverticulitis is “a sac-like protrusion” in the colonic mucosa that protrudes through the muscles of the colon wall and commonly presents visible anatomical alterations. In Westernized countries [13,14], their incidence and prevalence are estimated to increase as life expectancy increases [15] and as diagnostic techniques improve [16]. Colon diverticulosis is estimated to occur in approximately 17.5% of the general population [17], with 30% of people over 60 years of age and 70% of people over 80 years of age whose course is generally asymptomatic; only 4% of these patients will develop chronic diverticulitis, whose complications range from abscess formation to perforation, peritonitis, bleeding, or intestinal obstruction.

Colon adenoma is a benign polyp that develops in the lining of the colon and has been linked to lifestyle factors such as smoking, alcohol consumption, being overweight or obese, and consuming diets high in fat and red or processed meats but low in fiber. Although most adenomas are benign, some can progress into cancer. In these cases, the composition of the gut microbiome may be notably altered, with increases in *Bacteroides, Parvimonas*, *Bilophila*, and *Fusobacterium* species and decreases in *Ruminococcus, Bifidobacterium*, and *Streptococcus* species [18,19,20]. These microbiome changes may influence local immune responses and produce genotoxins, such as colibactin, along with microbiome-specific metabolites such as secondary bile acids and short-chain fatty acids, which may regulate tumor initiation and progression to the tumor stage [20,21,22,23,24]. The overall goal of this study was to identify dysbiosis of the gut microbiome and its association with the development of colonic conditions such as irritable bowel syndrome, uncomplicated diverticulitis, and tubular adenoma.

## 2. Materials and Methods

### 2.1. Sample Collection

An observational, comparative, prospective, and cross-sectional study was performed. The sample size was determined by convenience rather than randomization. A total of 55 biopsy samples were collected via colonoscopy. The inclusion criteria included patients over 18 years old seen at Juárez de México Hospital for cancer screening who, according to the treating physician’s diagnosis, tested negative for cancer and reported symptoms suggestive of the conditions analyzed (such as abdominal pain, nausea and vomiting, and changes in bowel habits such as constipation or diarrhea) during their medical consultation. The samples were stored at −20 °C until processing. Demographic data were also gathered. The exclusion criteria included samples from patients with a confirmed diagnosis of inflammatory bowel disease, colorectal cancer, intestinal infections (parasitosis, tuberculosis), systemic hormonal disorders, food intolerance, celiac disease, thyroid disease, bile acid malabsorption, enteric neuropathy or myopathy, or symptoms caused by medication side effects, and the elimination criteria included samples from patients who chose to withdraw informed consent, had incomplete clinical records, insufficient sample quantity, or failed amplification of the 16S rRNA gene.

### 2.2. Metagenomic DNA Extraction and Quality Control

Approximately 25 mg of tissue from each patient biopsy sample was used to obtain metagenomic DNA via the DNeasy Blood & Tissue Kit (QIAGEN, Venlo, The Netherlands). The DNA concentration and purity were quantified via a nanophotometer (Nanodrop). Metagenomic DNA integrity was visualized on 1% agarose gels, and amplification was corroborated by endpoint PCR assays of the complete 16S rRNA gene with the primers reported by DeSantis [25] 27F/1492R. The samples with positive gene amplification products were subsequently subjected to library construction and subsequent sequencing via Illumina MiSeq technology.

### 2.3. Amplification of the V3 and V4 Regions

The V3-V4 hypervariable region of the 16S rRNA gene was amplified via the Quick-16S Plus NGS Library Prep Kit (V3-V4, UDI) with 96-reaction Primer Set 3 (D6421-PS3) from Zymo Research, Irvine, CA, USA. Amplicons from these regions were purified with AMPure XP beads (Beckman Coulter, Brea, CA, USA). Libraries were sequenced via a 150X2 protocol on the Illumina MiSeq platform, and PhiX phage was used as a sequencing control, as recommended by the manufacturer.

### 2.4. Quality Control and Taxonomic Assignment

Illumina raw-free sequences were analyzed by using the QIIME2 amplicon version v2021.11 [26]. Quality control, chimera removal, and clustering of sequences into operational taxonomic units (OTUs) were performed with the DADA2 algorithm (Callahan et al., 2016) [27]. Taxonomic assignment was carried out by training QIIME2 with the SILVA v138 SSU database [28]. The results were filtered by removing unidentified sequences and duplicate sequences via a Python script. Rarefaction, alpha diversity, beta diversity, and downstream analyses were performed with Microbiome v3.21 and Phyloseq library v1.42.0 by using R v4.2. Alpha diversity was calculated via the Shannon–Wiener and Gini–Simpson indices. Conversely, beta diversity analysis was performed with the weighted and unweighted UniFrac method; the obtained matrices were obtained through NMDS (nonmetric multidimensional scaling) and PCoA (principal coordinate analysis).

The heatmap and taxonomic bar plots were constructed with the top 20 most abundant bacteria. All these analyses were plotted with the ggplot2 library and R v4.2.

### 2.5. Differential Presence Analysis and Random Forest

The calculation of the differential presence of microorganisms was performed via the DESeq2 v3.21 library in R v4.2, which is based on tables of relative abundance and taxonomic assignment. Microorganisms with a relative abundance higher than 2% were filtered out. The *p*-values of paired comparisons were corrected with the Bonferroni method. The random forest analysis was performed via the scikit-learn library version 1.71 with a Python script. For the training of the random forest algorithm, 1000 trees were constructed, and the mean decrease in Gini value was calculated as a metric for evaluating the importance of microorganisms.

Additionally, as a model validation, the ROC-AUC metric (receiver operating characteristic area under the curve) with a 10-fold cross-validation was calculated. For validation, the data were split into training and testing sets, with 80% allocated for training and 20% allocated for testing the random forest algorithm. The graphs were generated via R v4.2 and the ggplot2 library.

### 2.6. Statistical Analysis

For each disease, alpha and beta diversity analyses were conducted via R v4.2, and one-way ANOVA was applied to identify significant differences between clinical groups, with a *p*-value of less than 0.05. Differential presence analysis was performed with the DESeq2 v3.21 library. Demographic data are summarized as the means (standard deviations, SDs) for numerical variables and absolute counts (percentages) for categorical variables (Table 1). Categorical variables were compared via Fisher’s exact test. The chi-square test was applied to relate the type of intestinal pathology and the final outcome of the pathogenic and commensal populations. *p*-values < 0.05 were considered statistically significant. The 95% confidence intervals for proportions were calculated. All analyses were conducted via IBM SPSS Statistics for Windows, version 31 (IBM Corp., Armonk, NY, USA).

## 3. Results

### 3.1. Demographic Data

In total, 55 biopsy samples were obtained; however, only 45 were included. A total of 45 samples from patients with irritable bowel syndrome (27), uncomplicated diverticulitis (9), and tubular adenoma (9) were included. A total of 55.5% of the patients were female. No significant differences were observed between the groups in terms of sex or weight; however, significant differences were noted among the age groups. The detailed demographic data of the participants are summarized in Table 1.

### 3.2. Alpha Diversity and Rarefaction Analysis

**Figure 1:**
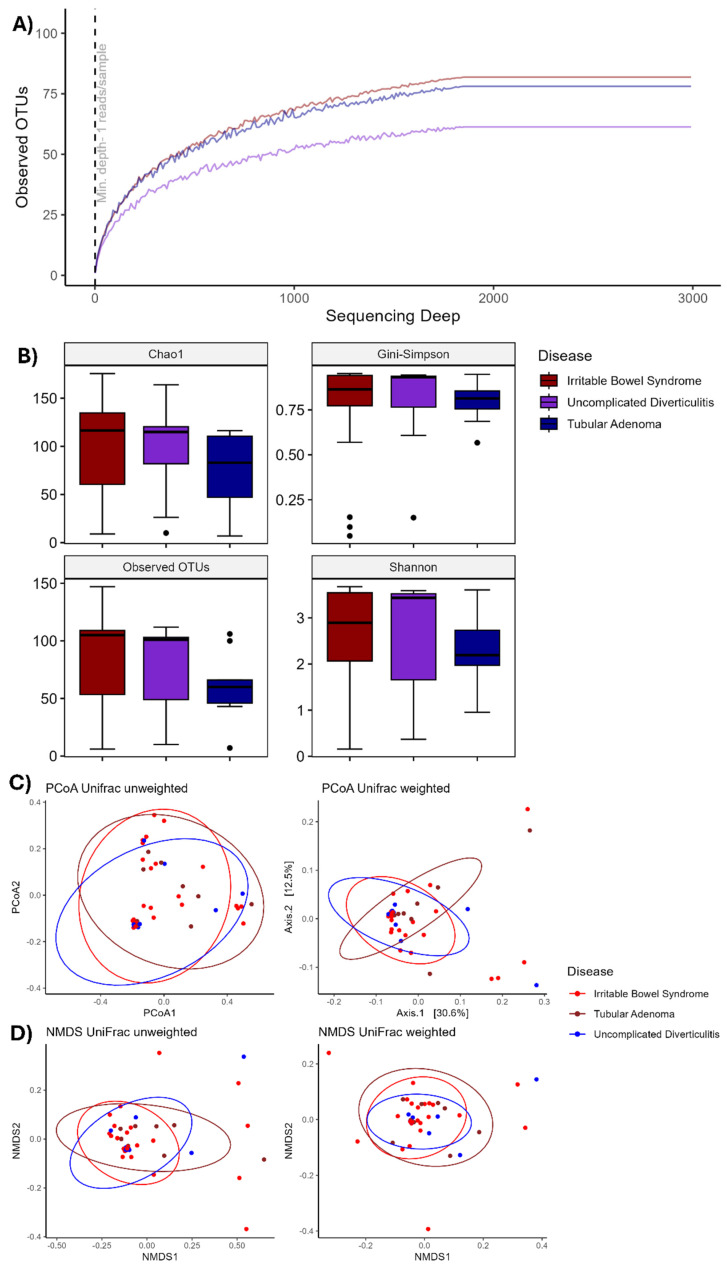
Analysis of the differences in the alpha and beta diversity. (**A**) Rarefaction analysis to evaluate a sufficient sample. (**B**) Analysis of the differences in the observed alpha diversity and Chao1, Shannon, and Gini–Simpson indices among patients with irritable bowel syndrome, uncomplicated diverticulitis, and tubular adenoma. There was a significant difference in each comparison (*p* < 0.001). (**C**) Beta diversity analysis via the weighted and unweighted UniFrac methods revealed no separation between the microbial populations present in samples from patients with irritable bowel syndrome, uncomplicated diverticulitis, and tubular adenoma. (**D**) Beta diversity analysis using the weighted and unweighted UniFrac methods revealed no separation between the microbial populations present in samples from patients with irritable bowel syndrome, uncomplicated diverticulitis, and tubular adenoma.

Before alpha diversity calculation, radiation analysis was performed to evaluate whether the sample data were sufficient to determine all the bacterial diversity in the samples (Figure 1A). The rarefaction plot indicated that our samples were adequate for observing complete bacterial diversity, as the OTUs in each clinical group stabilized after 1800 sequences were obtained. According to the rarefaction plot, alpha diversity assessed via the Chao1, Shannon–Werner, and Gini–Simpson indices varied across the three clinical conditions (Figure 1B). Tubular adenoma exhibited significantly lower diversity according to the Shannon and Chao1 indices (*p* < 0.001). A similar trend was observed when irritable bowel syndrome was compared with uncomplicated diverticulitis (*p* < 0.001). However, no significant difference in diversity was found between tubular adenoma and uncomplicated diverticulitis (*p* = 0.48), indicating a loss of diversity under more aggressive colonic conditions, implying changes in abundance within conditions and hence changes in the signature of the microorganisms present.

### 3.3. Beta Diversity Analysis

Beta diversity analysis revealed no significant difference (*p* = 0.1) between the clinical groups, as determined by both the weighted and unweighted UniFrac methods via PCoA (Figure 1C) and NDMS (Figure 1D). This result is interesting; hence, there are no distinct changes in the major composition of the microorganisms present in each disease (the bacteria found were almost similar in all the samples), indicating that there are only distinctive changes in the abundance of specific OTUs, as we observed in alpha diversity. These differences in the abundance of OTUs will be further investigated in the following sections.

### 3.4. Taxonomic Composition

**Figure 2:**
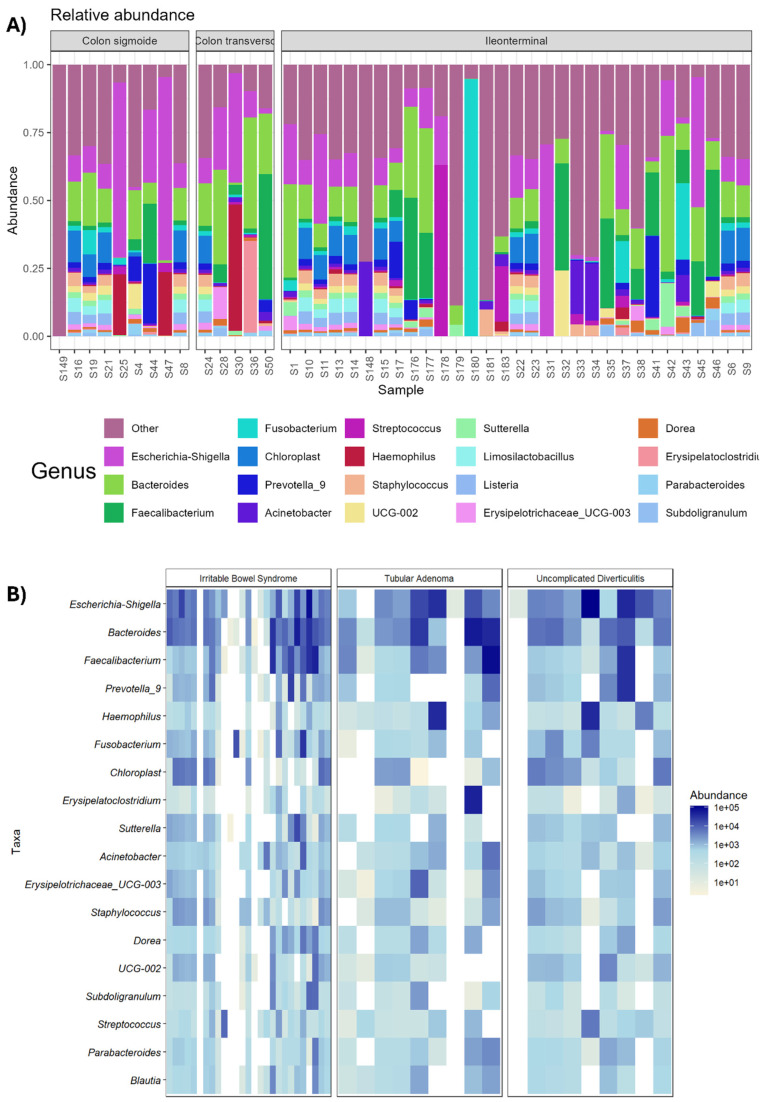
Analysis of taxonomic composition. (**A**) Relative abundance at the genus level of the total taxonomic composition of everyone included in the study in the samples from patients with irritable bowel syndrome, uncomplicated diverticulitis, and tubular adenoma. (**B**) Heatmap showing the differentially abundant genera among the three conditions expressed in log2. To explore specific taxonomic signatures at the level of OTUs among the different conditions, the datasets were hierarchically clustered via a dendrogram.

Once we observed the alpha and beta diversity, we examined the bacterial taxonomic composition and identified 642 different OTUs assigned to all the samples. These data are reported in a bar plot, with each group of bars denoting a clinical group, and the abundances were normalized to one and represented at the genus level for the top 20 most abundant OTUs. The taxonomic bar plot (Figure 2A) displays bacteria that may have an important niche within the disease, as well as several bacteria whose abundance changes within clinical groups. When the microbiomes of the samples from the three conditions were compared, as shown in Figure 2, the percentage abundances of the assigned genera Escherichia–Shigella and Fusobacterium changed noticeably. IBS exhibits high diversity in terms of alpha diversity.

### 3.5. Relative Abundance

Taxonomic profiling of the metagenome data revealed several bacteria with medical importance and highlighted the genera *Fusobacteria*, *Prevotella*, and *Haemophilus* with known pathogenic roles. A heatmap of the top 20 most abundant microorganisms was constructed (Figure 2B). This plot shows the differentially abundant genera between the three conditions expressed in log10 to visualize a better contrast in the abundance of each OTU. As we expected, all bacteria were present in the three clinical groups. However, these 20 genera decreased in abundance in tubular adenoma, followed by uncomplicated diverticulitis and irritable bowel syndrome, which are predominantly known commensal bacterial families and genera, such as *Catenibacterium*, *Bifidobacterium*, and *Faecalibacterium*.

Alternatively, the presence of the *Escherichia–Shigella* complex, also known as the enterotoxigenic genus Bacteroides, was observed under all three conditions. This heatmap helps us explore the specific taxonomic signatures at the OTU level between the different diseases. Interestingly, in several samples of tubular adenoma and uncomplicated diverticulitis, a high prevalence of *Haemophilus* was detected.

### 3.6. Differential Presence Analysis and Random Forest

**Figure 3:**
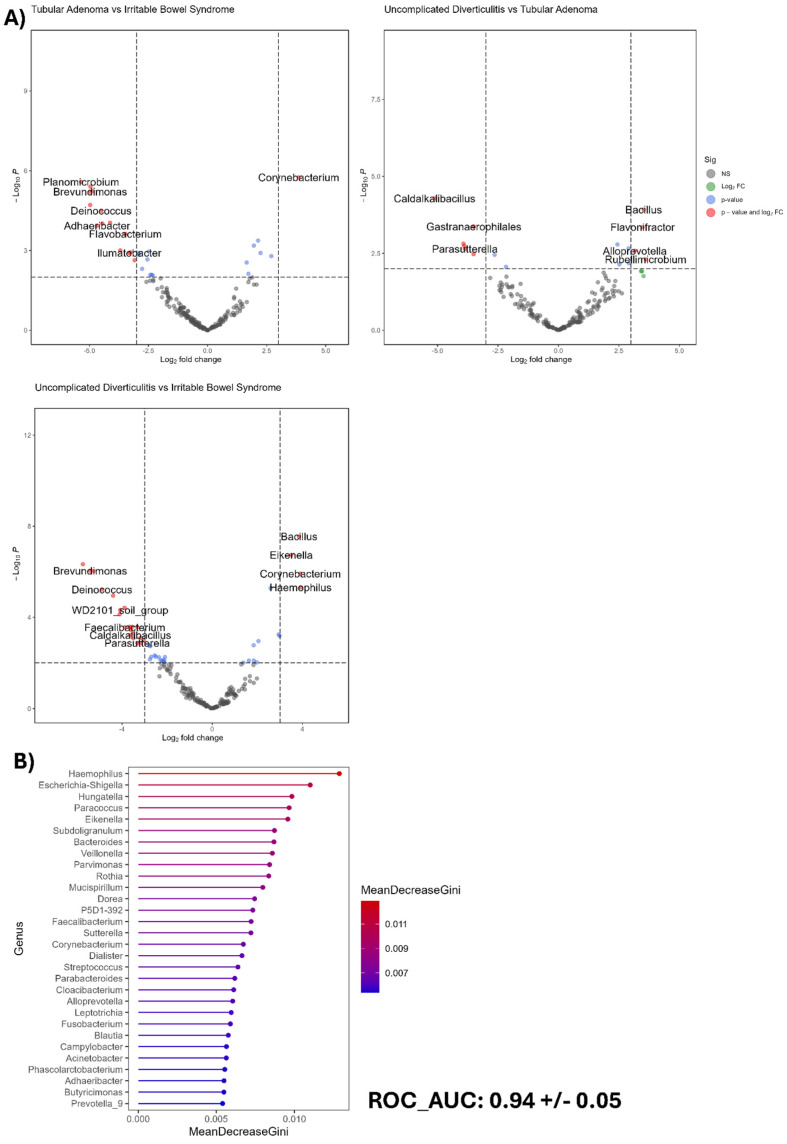
(**A**) Paired differential presence analysis showing the characteristic genera for each condition. (**B**) Random forest analysis revealed that *Haemophilus* and *Escherichia-Shigella* were among the most important bacterial genera.

After observing the beta diversity results, we found no differences, so we performed an analysis to identify which OTU abundance varied among the diseases assayed. A study was proposed to investigate the differential presence of microorganisms (Figure 3A), aiming to identify the bacteria that are more abundant within each clinical group. In our case, for IBS, the presence of the OTUs assigned to the genera *Haemophilus* and *Corynebacterium* was observed. In the case of uncomplicated diverticulitis, *Parasutterella* and *Caldalkabacillus* are mostly present. Finally, *Flavobacterium* is pathogenic under certain conditions in tubular adenoma. This finding highlights the distinctive ecological niches of some microorganisms under each condition.

On the other hand, to identify which OTUs may distinguish between the three clinical groups, we performed a random forest analysis to select the important features in our data for choosing OTUs capable of differentiating the disease groups. Additionally, three metrics were used to evaluate the ability of the random forest model to assess its performance (Figure 3B). The mean decreases the Gini (MDG) coefficient of the top 30 OTUs was calculated. Several bacteria identified were consistent with those identified in the differential analysis. Furthermore, the random forest model demonstrated its ability to select optimal features, as evidenced by the ROC-AUC metric, which had a value of 0.94 ± 0.05. The ROC-AUC metric measures the model’s ability to distinguish between each clinical group. In our case, we obtained a value of 0.94; in other words, variations in abundance among diseases are enough to identify the patterns in the data. These results indicate that the random forest model was well trained and may even be an excellent predictor of the observed abundances of OTUs in samples in the three clinical groups, even for detecting the bacterial signature in each clinical group.

To determine a possible association between dysbiosis and each condition, the chi-square test was used to analyze pathogenic and commensal microbial populations. No significant association (*p* = 0.32) was found between the type of pathology and the total pathogenic or commensal disease score (Table 2); however, a ratio of 2.54 (pathogenic to commensal) was observed in the IBS patient group. In contrast, in the diverticulitis and adenoma patient groups, this ratio was 8 (pathogenic and commensal), respectively.

## 4. Discussion

The colonic microbiome is dominated primarily by anaerobic bacteria, including thousands of species distributed among the different phyla that comprise it, and its persistence in the colon can be hampered by numerous factors and chemical parameters, such as pH, oxygen concentrations and redox potential; the biological production of mucus, bile and antibodies; and physical aspects, such as the intestinal architecture, peristalsis and host transit times. This study revealed distinctive compositional changes in the microbiome of irritable bowel syndrome patients compared with those of uncomplicated diverticulitis patients and tubular adenoma patients, with a greater diversity of OTUs observed in IBS patients. Additionally, the abundance of several taxa of known pathogens was greater in patients with tubular adenoma than in those with diverticulitis or IBS. The gut harbors a complex bacterial community consisting mainly of two bacterial phyla, Bacteroidetes and Firmicutes [29]. An alteration in their composition affects the balance of all colonic populations, favoring the presence and multiplication of pathogenic bacteria and, with this, alterations in the microhabitat, causing processes such as inflammation, altering the immune system, or allowing an abnormal response to potentially pathogenic bacteria, including the proliferation of these bacteria [30]. In this study, we observed a 4-fold increase in the pathogen/commensal ratio in patients with diverticulitis and adenoma. Future studies are needed to investigate the mechanisms that promote the increase in pathogenic populations over commensal populations in various colonic pathologies.

Recently, some microorganisms, such as *Fusobacterium nucleatum*, *Enterococcus faecalis*, *Streptococcus bovis enterotoxigenic Bacteroides fragilis* (ETBF), and *Porphyromonas* spp., have been shown to play essential roles in the pathogenesis of adenoma and other conditions, such as *Roseburia* spp., *Eubacterium* spp., *Lactobacillus* spp., and *Bifidobacterium* spp. [31,32,33]. In the present study, an increase in the *Escherichia-Shigella* group was observed under all three conditions studied. *Shigella* is classified as a Gram-negative intracellular bacterial pathogen that initiates infection by invading cells, causing intense inflammation in the colonic and rectal epithelium, which has been associated with malignancy and an increased risk of colorectal cancer [34]. An increase in the enterotoxigenic genus *Bacteroides*, which has been proposed as a pathogen in adenoma malignancy [35], an increase in the genera *Fusobacteria*, *Prevotella*, and *Haemophilus*, and a decrease in the commensal bacterial genera *Catenibacterium*, *Bifidobacterium*, and *Faecalibacterium*, which are involved in food decomposition, nutrient absorption, and control of pathogenic microorganisms [36], were also observed.

These variations in the composition of the colonic microbiome have also been reported in other conditions, such as inflammatory bowel disease (IBD) [37] and metabolic syndrome [38,39,40]. Prototypical inflammatory disorders of the gut have been associated with a deviated gut microbiome composition. Indeed, an increase in facultative anaerobes has been reported, especially in the context of active inflammation and alterations in metabolites, including short-chain fatty acids (SCFAs) and acylcarnitine pathways. Additionally, specific bacterial species have been identified as infiltrating the epithelium and submucosa in patients with acute appendicitis [41].

In the case of the genus *Haemophilus*, multiple studies have reported that intestinal colonization by *Haemophilus parainfluenzae*, an oral commensal, is associated with IBD activity [42,43,44]. Indeed, the presence of *H. parainfluenzae* in intestinal mucosal tissue was one of the most influential predictors of disease activity in treatment-naive patients with Crohn’s disease [45]. Similarly, *H. parainfluenzae* is strongly associated with severity, progression, and response to treatment in ulcerative colitis (UC), another critical IBD subtype [46]. Future studies are needed to identify the *Haemophilus* species detected and their associations with the conditions studied here, as well as with the *Escherichia-Shigella* group.

Barbara et al. [47] studied the microbiome in uncomplicated symptomatic diverticular disease and reported a decreased abundance of *Clostridium* cluster IX, *Fusobacterium*, and *Lactobacillaceae* compared with that in controls. In a second study, an increased abundance of *Akkermansia muciniphila* was observed in patients with asymptomatic diverticulosis compared with controls.

These pathological states related to changes in the microbiome arise through two mechanisms: first, through the presence (or increased abundance) of the disease-causing microbiome and second, through a decrease in the abundance and reduced diversity of the commensal microbiome [48]; however, there is still controversy as to whether these changes are disease-causing or simply a phenotype of the pathological process itself. Overall, this study describes the differences in the abundance of intestinal microbiota in three colonic pathologies and the increase in pathogenic microbial populations compared to commensal populations. A structural imbalance in the gut flora composition is characterized by a decrease in beneficial bacteria and an increase in harmful bacteria. The presence of certain microbial genera in each group suggests the potential for their use as disease-specific biomarkers. Our findings indicate that further research is necessary to investigate the prevalence of *Haemophilus*, *Escherichia*, *Shigella*, and *Bacteroides* and the mechanisms associated with their increase and how this influences the cellular rearrangement of the colon, as well as their association with these conditions, which could confirm their potential as promising biomarkers for early detection. However, a larger sample size is needed to validate the candidate biomarkers further.

The intestinal bacterial community plays a crucial role in regulating various aspects of metabolic disorders. This regulation depends, among other things, on the production of a wide variety of metabolites by the microbiota and its interactions with receptors on host cells, which can activate or inhibit signaling pathways and have either beneficial or detrimental effects on host health. Therefore, an alteration in the composition of microbial communities can affect the entire colonic environment.

## 5. Limitations

Limitations of this study include the small sample size and the fact that analyzing a single time point offers only a snapshot. The observed microbiome changes may not accurately reflect an individual’s long-term microbiome composition and could be responses to inflammatory or pathological processes rather than contributors to overall disease development. Additionally, the samples were from individuals undergoing screening colonoscopies, which may introduce bias due to the effects of mechanical bowel preparation (MBP) on the microbial composition. Another limitation arises from 16S rRNA gene sequencing, since this technique only amplifies two regions of the nine hypervariable regions of the 16S rRNA gene, providing information mostly at the genus level and potentially influenced by the initial DNA extraction method. While this remains a challenging area of research, gaining a better understanding of the microbiome’s potential role in the development of the three conditions discussed here is clinically important and warrants further study.

## 6. Conclusions

These results provide evidence supporting the proposal that alterations in the colonic microbiome could be involved in various colonic pathogeneses and that an imbalance between commensal and pathogenic populations could be directly related to pathogenesis in the microsystem. It is important to highlight the need for future studies.

## Figures and Tables

**Table 1:** Demographic and disease characteristics of the included individuals. Max: maximum; Min: minimum; SD: standard deviation.

	IBS	Diverticulitis	Tubular Adenoma	*p* Value
AGE				0.033 *
Mean (SD)	47.7 (16.9)	50.2 (14.2)	64 (11.04)	
Median [Min, Max]	53 [10, 85]	55.5 [22, 68]	65 [44, 77]	
GENDER				0.062
Male, n (%)	10 (37%)	3 (33.3%)	6 (66.7%)	
Female, n (%)	17 (63%)	6 (66.7%)	3 (33.3%)	
WEIGHT, kg				0.117
Mean (SD)	72.3 (11.6)	69.44 (20.4)	79.8 (16.9)	
Median [Min, Max]	71 [53, 101]	67 [47, 115]	86 [57, 103]	

* Fisher’s exact test.

**Table 2:** Relationship between the type of intestinal pathology and the relative abundance of pathogens.

	Intestinal Disease			Total
	IBS (n = 27)	Diverticulitis (n = 9)	Tubular Adenoma (n = 9)	
Pathogenic población	70.4% (19)	88.9% (8)	88.9% (8)	77.8% (35)
Commensal población	29.6% (8)	11.1% (1)	11.1% (1)	22.2% (10)

## Data Availability

The data from this study are shared through the following link: https://drive.google.com/drive/folders/1yrV1-EbP0qzcapp2D9kdwfsfKbZJyuM6?usp=sharing accessed on 1 October 2025.

## Abbreviations

The following abbreviations are used in this manuscript:

rRNA	Ribosomal ribonucleic acid
IBS	Irritable bowel syndrome
DNA	Deoxyribonucleic acid
PCR	Polymerase Chain Reaction
NMDS	Nonmetric multidimensional scaling.
ROC-AUC	Receiver operating curve
OTU	Operational Taxonomic Unit
NMDS	Nonmetric Multidimensional Scaling
PCoA	Principal Coordinates Analysis
IBD	Inflammatory bowel disease
MBP	Mechanical bowel preparation
MDG	Mean Decrease in Gini
